# Interrelations between clinical-psychological features and bone mineral density changes in post-menopausal women undergoing anti-osteoporotic treatment: a two-year follow-up

**DOI:** 10.3389/fendo.2023.1151199

**Published:** 2023-05-09

**Authors:** Gabriella Martino, Federica Bellone, Carmelo Mario Vicario, Agostino Gaudio, Francesco Corica, Giovanni Squadrito, Trine Lund-Jacobsen, Peter Schwarz, Gianluca Lo Coco, Nunziata Morabito, Antonino Catalano

**Affiliations:** ^1^ Department of Clinical and Experimental Medicine, University Hospital of Messina, Messina, Italy; ^2^ Department of Cognitive Sciences, Psychology, Education and Cultural Studies, University of Messina, Messina, Italy; ^3^ Department of Clinical and Experimental Medicine, University Hospital of Catania, Catania, Italy; ^4^ Department of Endocrinology, Centre for Cancer and Organ Diseases, The Copenhagen University Hospital, Rigshospitalet and Faculty of Health Sciences, University of Copenhagen, Copenhagen, Denmark; ^5^ Department of Psychology, Educational Science and Human Movement- University of Palermo, Palermo, Italy

**Keywords:** clinical psychology, anxiety, depression, quality of life, post-menopausal, osteoporosis, fracture, bisphosphonates

## Abstract

**Introduction:**

Psychological features have been bidirectionally associated with osteoporosis, but it is still unclear whether patient’s anxiety fluctuations during the anti-osteoporotic treatment can have an impact on bone mineral density (BMD) variation. The aim of this study was to investigate the interrelations between psychological distress features, such as anxiety, depression, health-related QoL (HRQoL) and bone health in women receiving anti-osteoporotic treatment.

**Methods:**

192 post-menopausal osteoporotic women were treated with alendronate or risedronate according to the standard procedure. The levels of anxiety, depression, and perceived HRQoL, along with BMD, were assessed at baseline and at a 2-year follow-up.

**Results:**

At the end of the study, the patients showed a statistically significant increase of both psychic and somatic anxiety (p<0.0001) and exhibited a worsening of depressive symptoms (p<0.0001), whereas HRQoL showed no change. BMD improved and no incident fractures occurred. BMD variation (ΔBMD) at lumbar spine was significantly associated with anxiety levels (r=0.23, p=0.021). Multiple regression analysis showed that both patients’ worsening anxiety levels (β = -0.1283, SE=0.06142, p=0.04) and their treatment adherence (β=0.09, SE=0.02, p=0.0006) were independently associated with ΔBMD.

**Discussion:**

The findings of the current follow-up study suggest that BMD in post-menopausal women undergoing anti-osteoporotic treatment was predicted by treatment adherence and anxiety change over time.

## 1 Introduction

Among chronic diseases, osteoporosis is a well-recognized major public health concern leading to an increased risk of bone fractures in post-menopausal women. The global burden of osteoporosis is expected to increase worldwide (1, 2). It is estimated that one out of three patients over fifty will suffer from at least one osteoporotic fracture during his/her lifetime. Fragility fractures, and especially major osteoporotic fractures, are strongly associated with pain, disability and even mortality (3). Among the Italian population, the prevalence of osteoporosis amounts to 6.3%, and women represent about 80% of all cases. Furthermore, the prevalence is expected to increase from 133,000 in 2019 to 702,000 in 2034 (3).

There is a growing interest regarding the key role of clinical psychological features in chronic diseases. Psychological factors may indeed contribute to predict chronic illness, due to affective and personality characteristics, as well as to adaptive strategies (4–12). For example, it was evidenced that psychological distress may be associated with pain, changes in people’s life and disease management, and that, vice versa, chronic conditions may cause psychological suffering and distress, also influencing the individual’s health-related quality of life (HRQoL) (13–16).

Anxiety disturbances represent one of the most frequent mental diseases and a leading cause of burden worldwide, with a consequent impact on important areas of life functioning (17). Anxiety diseases have a global estimated prevalence of 7.3% and they increased by 50% from 1990 to 2019 (18).

Anxiety is found to be a predictor of increased fracture risk, low BMD and fracture occurrence in post-menopausal women probably due to immunological and endocrine mechanisms (19–27). Several studies have shown the association between inflammatory markers and occurrence of anxiety symptoms (27). Accelerated bone turnover, i.e. increased bone resorption and reduction of bone formation due to inflammation, may raise the risk of bone fragility fractures as reported in chronic rheumatic inflammatory diseases (28). Moreover, as distressful conditions occur, higher levels of cortisol are shown in anxious patients, which leads to both a higher bone turnover and an accelerated loss of BMD (21–24). Furthermore, oxidative stress has been reported as a common pathogenic factor for both anxiety and osteoporosis risk (25, 26).

Several research studies have confirmed the positive role of both compliance and adherence to treatment in reducing morbidity and mortality, regardless of several confounders (29–31). Psychological distress features, such as anxiety and depression, are associated with socio-economic factors, which may also affect adherence to treatment, as recently reported by the World Health Organization. This is particularly highlighting the role of anxiety in identifying patient’s adherence and is suggesting its relevance in osteoporotic management (32).

Lately, it has been described that standard recommended anti-resorptive osteoporotic medications might be associated with a higher rate of psychopathological symptoms, such as anxiety and depression (33).

It was previously reported that anxiety levels are significantly associated with BMD and that anxiety levels are predictors of BMD loss (19, 20). However, it is still unknown whether anxiety levels change over time in post-menopausal women receiving standard antiresorptive medical treatment for osteoporosis.

The aim of the present study was to examine the association between anxiety and BMD in post-menopausal women during a 2-year follow-up study.

## 2 Materials and methods

### 2.1 Participants

At baseline, 192 Caucasian osteoporotic post-menopausal women who were referred to the Outpatient Clinic for the Prevention and Treatment of Metabolic Bone Disease, at the Department of Clinical and Experimental Medicine, University Hospital of Messina, Italy, have been enrolled between January 2017 and April 2017 (19). They have been evaluated from clinical, psychological and physic perspectives and, accordingly to the diagnosed osteoporotic disease, they received a weekly anti-osteoporotic prescription for either alendronate or risedronate at the dose of 70 mg and 35 mg, respectively.

After a two-yearfollow-up, 102 participants completed the studyfor both clinical psychological and physical evaluations.

At baseline and at the end of the follow-up, the recruited women were evaluated for both clinical psychological factors and physical parameters with a special focus on anxiety, depression, HRQoL, and BMD respectively. Adherence to antiresorptive treatment was reported during the clinical interview. Exclusion criteria were: no current or prior history of neurological or psychopathological disorders, according to DSM-5 criteria (34), present or previous physical diseases (e.g., liver, kidney, respiratory or heart failure; thyroid, parathyroid, or adrenal glands diseases, intestinal malabsorption, cancer) (19). During the follow-up all participants took a cholecalciferol dose of 25,000 IU, once every 14 days, to prevent vitamin D hypovitaminosis, which is in line with the national recommendation (35). This research was carried out in accordance with the relevant guidelines and regulations and its experimental protocol was approved by the local Ethical Committee “Gaetano Martino” of the University Hospital of Messina, Italy. All participants gave their written informed consent.

### 2.2 Psychological and physical assessment

All subjects were assessed by clinical psychologists in collaboration with physicians, in a confidential setting, accordingly to the gold standard of clinical psychological interviews and good clinical practices. The general recorded data were: Education, employment status, clinical risk factors for osteoporosis and/or fractures including age, body mass index (BMI), prevalent fragility fracture (except traumatic head, hand, and foot fractures), family history of hip fracture, smoking habits, alcohol intake, rheumatoid arthritis, use of glucocorticoids and presence of other causes of secondary osteoporosis, all intended as dichotomous variables. These variables allowed, through a computer-based algorithm (FRAX^®^, http://www.shef.ac.uk/FRAX, accessed at the time of recruitment) (36), to evaluate the probability of major osteoporotic and hip fractures within the next 10 years. At baseline and after a 2-year follow-up, the levels of anxiety, depression and HRQoL were assessed through a gold standard psychological interview (20). The Hamilton Anxiety Rating Scale (HAM-A) was administered to detect the entity of anxious symptoms, through the scoring of 14 items (assessed from 0, not present, to 4, severe) which are based on psychological and somatic symptoms (anxiety, tension, fear, insomnia, intellectual impairment, depression, somatic symptoms, sensory, cardiovascular, respiratory, gastrointestinal, genitourinary, autonomic diseases, and observed behavior during the interview. The Beck Depression Inventory second edition (BDI-II) was used to measure depressive symptoms, through 21 items scored from 0 to 4 points for every self-administered item (37). The Short Form-36 (SF-36) questionnaire was used to evaluate the perceived health-related quality of life. It was scored from 0 to 100 (100 represents excellent perceived QoL) through the following eight dimensions: physical functioning, social functioning, role limitation due to physical and emotional problems, mental health, pain, vitality, and perceived general health (38). SF-36 is largely administered to evaluate the affliction of several illnesses, also exploring the possible influence of treatment interventions (39).

At baseline and after two years, BMD values were measured by the gold-standard Dual-energy X-ray Absorptiometry (DXA) in antero-posterior projection at the lumbar spine (L1–L4) and femoral neck, using a Hologic Discovery Wi densitometer with an *in vitro* 0.5% coefficient of variation (40). Vertebral fractures were assessed through the spine X-ray examination at dorsal and lumbar sites. The diagnosis of vertebral fracture was made when the vertebral body showed a minimum height reduction of 20% in the matched anterior, middle, or posterior height in comparison with the same or the adjacent vertebra (41).

### 2.3 Statistical analysis

Statistical analyses were performed using MedCalc software (version 20.113). Particularly, the Kolmogorov-Smirnov test was applied to verify the normal distribution of values. The paired t-test was performed to test the null hypothesis, i.e. that the average of the difference between paired observations was zero. Regression analysis allowed exploring the degree of association between two variables. Multiple regression analyses investigated the relationship between a dependent variable and one or more explanatory variables. Particularly, a lumbar BMD change was considered as a dependent variable while age, BMI, baseline lumbar BMD, FRAX score for major osteoporotic fractures, baseline anxiety, anxiety changes and adherence to treatment were considered as independent variables.

The statistical significance for all the performed tests was considered for a *p-value* < 0.05.

## 3 Results

**Figure 1:**
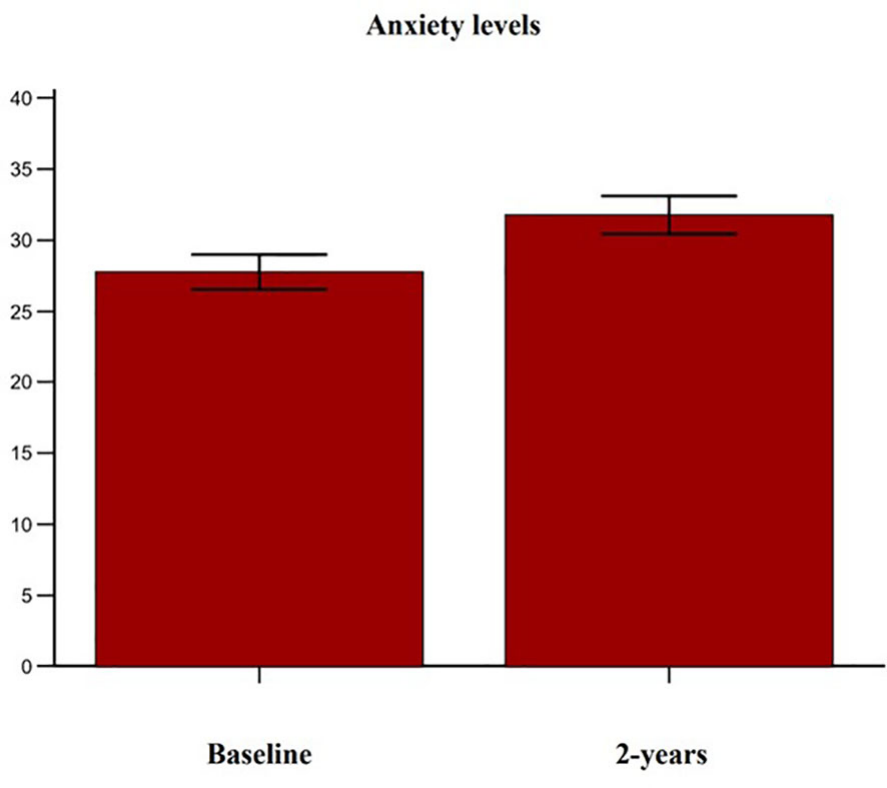
Variation of anxiety levels in accordance with the Hamilton Anxiety Rating Scale (HAM-A) score in osteoporotic postmenopausal women receiving oral bisphosphonates (alendronate or risedronate).

**Figure 2:**
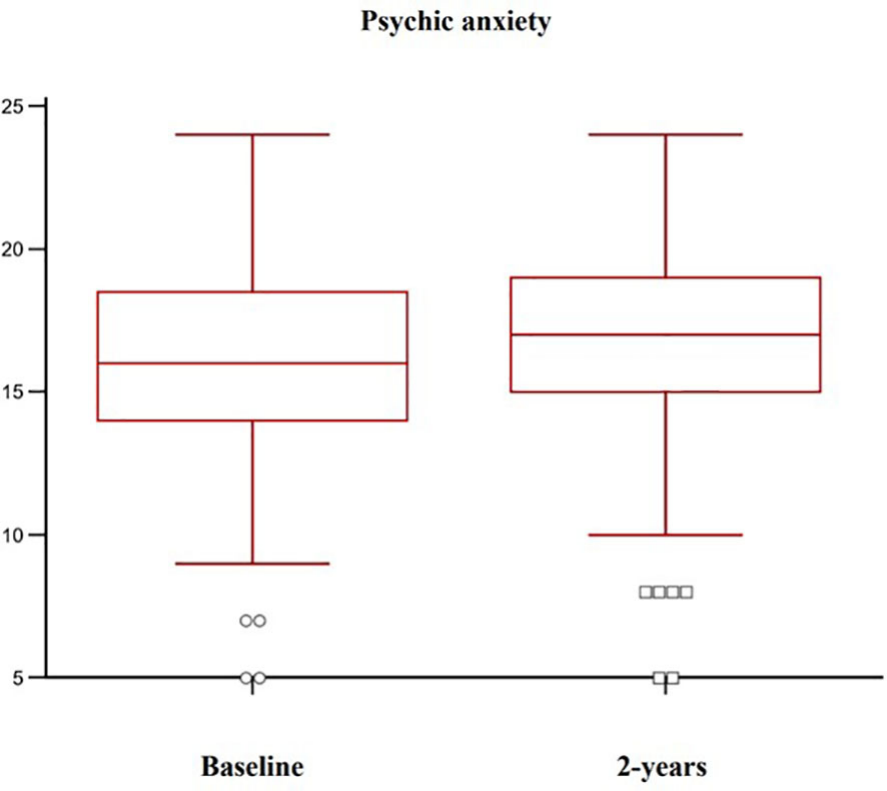
Variation of psychic anxiety levels in osteoporotic postmenopausal women receiving oral bisphosphonates (alendronate or risedronate).

**Figure 3:**
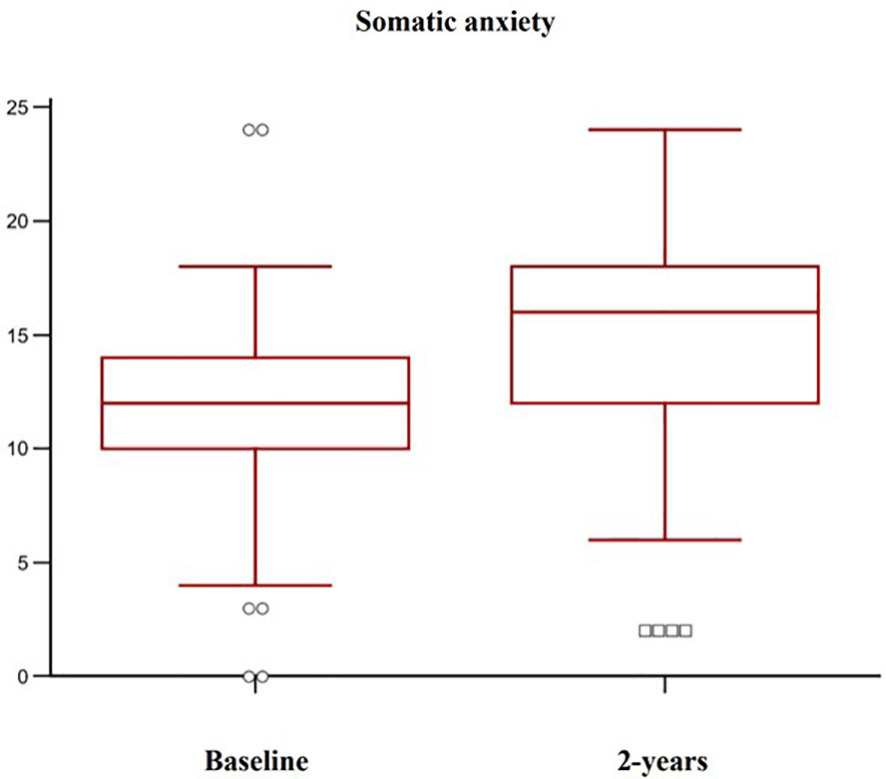
Variation of somatic anxiety levels in osteoporotic postmenopausal women receiving oral bisphosphonates (alendronate or risedronate).

**Figure 4:**
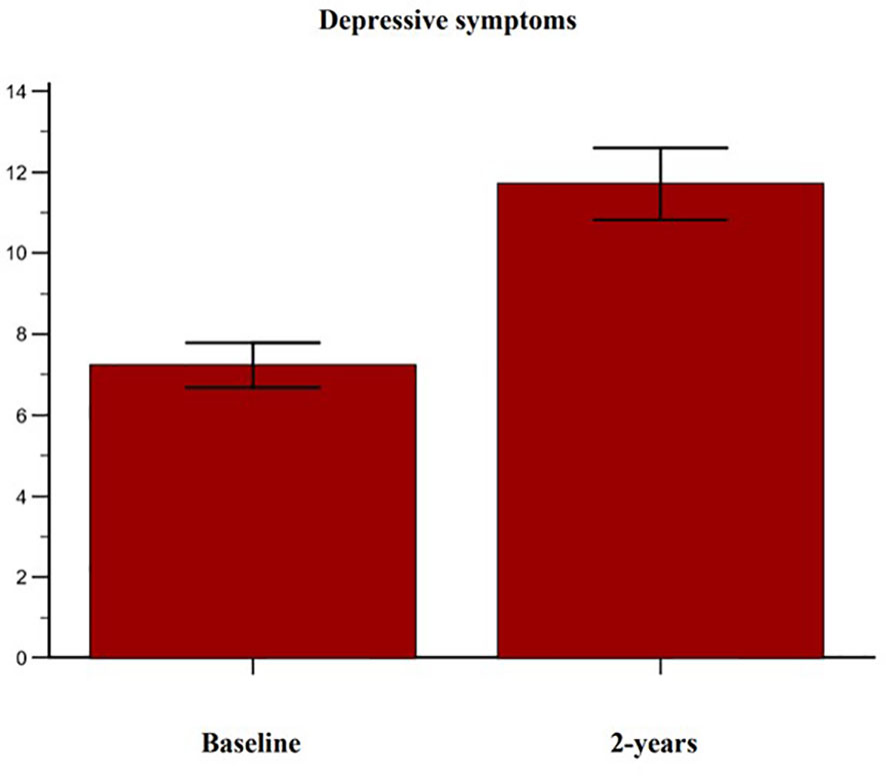
Variation of depressive symptoms in accordance with the Beck Depression Inventory- second edition (BDI-II) in osteoporotic postmenopausal women receiving oral bisphosphonates (alendronate or risedronate).

**Figure 5:**
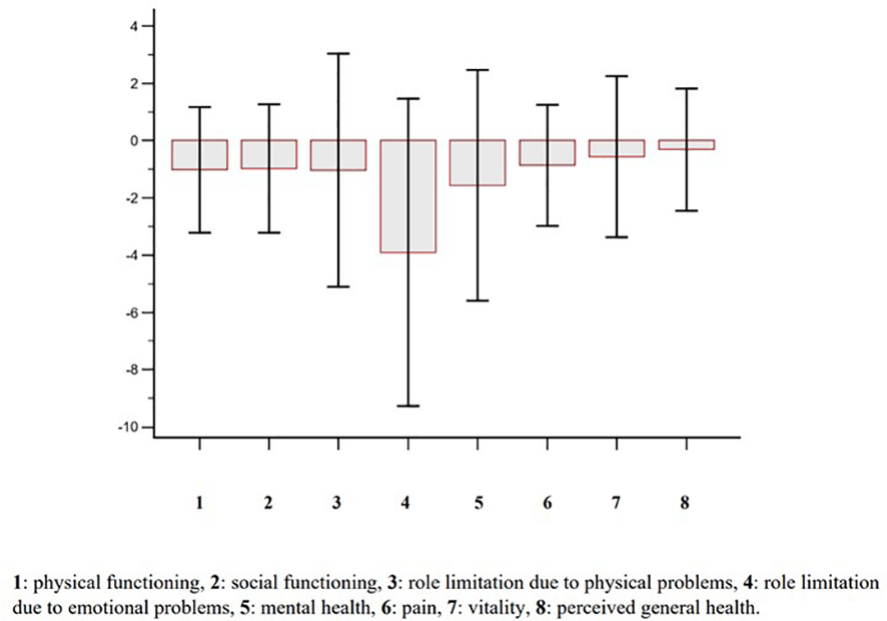
Change (2-yr vs baseline) of perceived health-related quality of life according with the Short Form-36 (SF-36) questionnaire in osteoporotic postmenopausal women receiving oral bisphosphonates (alendronate or risedronate).

**Figure 6:**
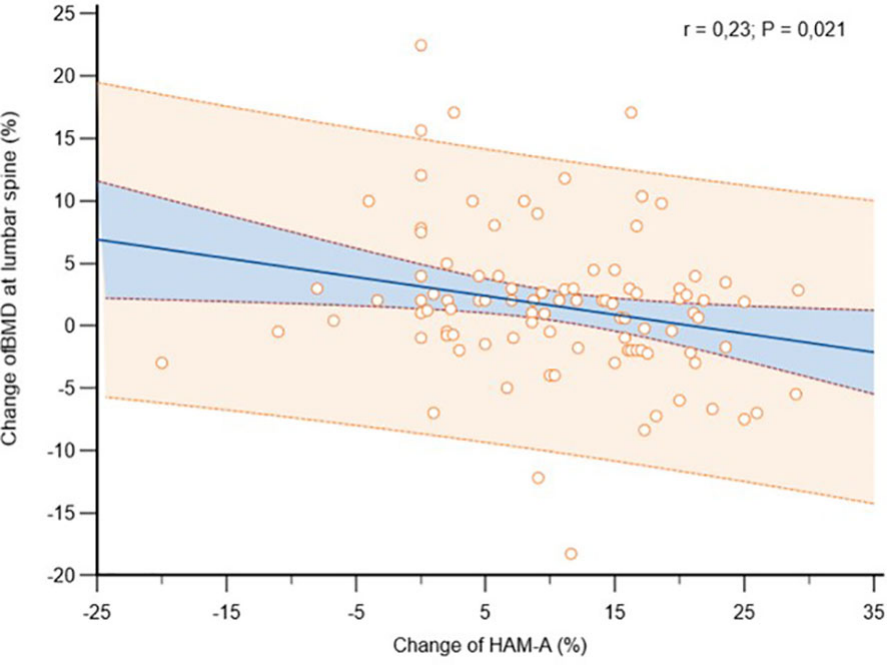
Association between anxiety levels change, measured in accordance with the Hamilton Anxiety Rating Scale (HAM-A), with bone mineral density (BMD) change at lumbar spine, in osteoporotic postmenopausal women receiving oral bisphosphonates (alendronate or risedronate).

Complete follow-up data at 2 years were available for 102 post-menopausal women. Their main clinical baseline characteristics are shown in **Table 1**, reporting a mean age of 67.5 years, a mean BMI in the overweight range, and a high prevalence of prior fragility fractures, with a high estimated 10-year fracture risk. Moreover, participants reported moderate to severe anxiety symptoms and low levels of depressive symptoms at baseline. At follow-up, patients showed a statistically significant increase of anxiety at HAM-A (27.7 ± 6.9 vs 31.7 ± 7.6, p < 0.0001) (**Figure 1**). Specifically, both psychic and somatic anxiety significantly increased (15.9 ± 3.8 vs 16.8 ± 4 and 11.8 ± 3.9 vs 14.9 ± 4.7 respectively; p^all^ <0.0001, see **Figures 2**, **3**). At the same time, patients exhibited a worsening in depressive symptoms (7.23 ± 3.16 vs 11.7 ± 5; p < 0.0001, **Figure 4**), whereas there was no change in the perceived QoL (see **Figure 5**). Moreover, the patients showed a significantly improved bone health, i.e. an improved BMD (0.8 ± 0.11 vs 0.83 ± 0.1, p = 0.0001, at lumbar spine; and 0.63 ± 0.08 vs 0.65 ± 0.09, p = 0.1, at femoral neck). None of the patients reported clinical fractures during the follow-up, as shown during the complementary clinical and instrumental evaluations. A univariate regression analysis shows that lumbar BMD variation (ΔBMD) was significantly and positively associated with anxiety levels (r = 0.23, p = 0.021; **Figure 6**), but not with depressive symptoms. On the other hand, femoral ΔBMD was not significantly associated nor with anxious neither with depressive symptoms (r = - 0.12, p = 0.38; r = - 0.13, p = 0.43; respectively). In the first model of multiple regression analysis, lumbar ΔBMD was considered as a dependent variable while anxiety changes (ΔHAM-A), age, BMI, baseline lumbar BMD, and FRAX score for major osteoporotic fractures were considered as independent variables, revealing that only ΔHAM-A independently predicted lumbar BMD modifications over time (β = -0.1497, SE = 0.06537, p = 0.02). Moreover, through a second multiple regression modelalso including baseline anxiety and adherence to treatment, it has been highlighted that only ΔHAM-A (β = -0.1283, SE = 0.06142, p = 0.04) and adherence (β = 0.09, SE = 0.02, p = 0.0006) were independently associated with ΔBMD at lumbar spine.

**Table 1:** Main clinical features of participants.

	Total (n=102)
Risk factors for osteoporosis	
**Age *(yr)***	67.5 ± 9.6
**Age at menopause *(yr)***	47.5 ± 5.9
**Time since menopause *(yr)***	20.6 ± 9.1
**BMI *(Kg/m^2^)***	24.9 ± 5.1
**Previous fracture *[n(%)]***	89 (87.5)
**Parent fractured hip *[n(%)]***	46 (45.3)
**Smoking habit *[n(%)]***	17 (16.6)
**Glucocorticoids *[n(%)]***	5 (4.9)
**Reumathoid arthritis *[n(%)]***	2 (1.9)
**Secondary osteoporosis *[n(%)]***	28 (27.4)
**Alcohol ≥ 3units/day *[n(%)]***	0
Education	
**Primary school *[n(%)]***	10 (9.8)
**Secondary school *[n(%)]***	17 (16.6)
**High school *[n(%)]***	38 (37.2)
**Bechelors’s degree *[n(%)]***	20 (19.6)
**PhD or specialization *[n(%)]***	17 (16.6)
Employment status	
**Housewife *[n(%)]***	21 (20.6)
**Full time *[n(%)]***	16 (1.6)
**Unemployed *[n(%)]***	3 (2.9)
**Pensioner *[n(%)]***	62 (60.7)
10-yr probability of fractures	
**Major osteoporotic fractures *[%]***	21.1 ± 10.8
**Hip fracture *[%]***	7.2 ± 8.9
Bone mineral density (BMD)	
**L1-L4 BMD *[gr/cm^2^]***	0.80 ± 0.15
**L1-L4 T-score *[SD]***	-2.15 ± 1.17
**Femoral neck BMD *[gr/cm^2^]***	0.63 ± 0.09
**Femoral neck T-score *[SD]***	-1.9 ± 0.84
Anxiety levels	
**HAM-A score**	27.7 ± 6.9
**HAM-A somatic symptom score**	11.8 ± 3.9
**HAM-A psychic symptom score**	15.9 ± 3.8
Depression severity	
**BDI-II score**	7.2 ± 3.1

HAM-A, Hamilton Anxiety Scale; BDI-II, Beck Depression Inventory-second edition.

## 4 Discussion

To our knowledge, this is the first longitudinal study that evaluated the association between anxiety and BMD changes in post-menopausal women undergoing medical treatment for osteoporosis. At the end of the study, it was observed anxiety symptoms significantly influenced BMD variations over time and that both psychic and somatic anxiety concurred to this outcome.

These data enhance the awareness concerning the association between fracture risk and clinical psychological symptoms and disorders (42–45). Among the clinical psychological features, anxiety is one of the most frequent impact causes on the crucial functioning areas and QoL.

Anxiety, in some particular facets, is one of the most frequent mental disorders and it is largely represented in patients with chronic diseases (18, 46).

Previous studies have observed a significant association between higher anxiety levels and poor bone health in general population (47, 48) and even in post-menopausal women with osteoporosis (19, 20), probably due to some common pathophysiologic factors underlying these two pathologies (21–27).

Higher anxiety levels could also contribute to the development of an adequate adherence to medical treatment in post-menopausal osteoporotic women (20). It is known that a lower adherence exposes patients to a poorer treatment response and to a worst BMD (49).

It is interesting to consider that post-menopausal women’s adherence is generally sub-optimal, especially in the primary care settings (50), while it is higher in post-menopausal women who are in a regular follow-up at osteoporotic specialized centers. Additionally, higher education levels and a deeper consciousness relative to chronic conditions drive patients to assume the prescribed drugs during the follow-up. Nevertheless, patients with higher education levels showed higher anxiety symptoms, suggesting anxiety could be a reliable adherence marker (20).

This study focuses on anxiety changes over time and shows increased mean anxious symptoms at the follow-up. In addition, beyond the predictive role of basal anxiety on adherence and response to treatment, this study highlights the influence of anxiety modification on BMD gain over the two-year observational period. Particularly, an intensification of anxiety over time blunted the response to bisphosphonates treatment, leading to a poorer BMD improvement. This could be due at least in part to endocrine and immune factors, which are involved in anxiety pathogenesisand also influence bone metabolism (21–27). It could be possible that bisphosphonate treatment leads to clinical psychological effectssuch as anxiety and depression since bisphosphonates cross the blood-brain barrier and may suppress the activity of acetylcholinesterase enzymes at the frontal cortex level (51). Consistently, Schmidt et al. reported that alendronate is able to inhibit the protein-tyrosine phosphatase (PTP) activity (52) whose physiological role has been well established in the development of the nervous systemand of the pituitary gland. Furthermore, it has a role in spinal cord injury and repair (53) and regulates osteoclast formation and function (52). This evidence doesn’t allow a more accurate clarification of the mechanisms involved in psychological clues, thus further studies on this topic are needed.

With regard to the QoL it is known that, in a 10-yr prospective population-based study, osteoporosis diagnosis has been associated with the worst perceived QoL, independently from its clinical manifestations (i.e. fractures), in comparison with women without osteoporosis, possibly due to several factors as fragility *per se*, perception about the disease itself, fear of falling, stress due to lack of certainty (54). In the current study, the perceived HRQoL was substantially unchanged over time, while its worsening could have been hypothesized accordingly to both higher anxiety and age and in line with previous observations (54). Therefore, it could be speculated that the BMD improvement along with the fracture and pain reduction may have protected women from a QoL worsening at the follow-up and that the mandatory vitamin D supplementation may have contributed as well (55–57).

We acknowledge that this investigation has some limitations such as the enrolment of only post-menopausal women, the small sample size, and the follow-up not being long enough to cover the long-term osteoporosis management. At the same time, the strengths are represented by the homogeneous group of participants, the clinical psychological interview and investigation and the standard osteoporotic treatment applied.

In conclusion, this longitudinal study highlights that osteoporotic post-menopausal women receiving oral bisphosphonates showed higher anxiety and depressive symptoms over time, without significant change in HRQoL. The patients also presented a BMD improvement which was associated with both adherence and anxiety changes, independently from other confounders. Particularly, on the one hand anxiety levels predicted adherence and allowed to identify patients with a fruitful response to medical treatment and on the other hand higher anxiety changes were associated with a lower BMD improvement. Further researches on this topic are needed to deeply comprehend the link between clinical psychology and bone health in order to build a specific gold-standard integrated clinical approach.

## Data availability statement

The raw data supporting the conclusions of this article will be made available by the authors, without undue reservation.

## Ethics statement

The studies involving human participants were reviewed and approved by Ethical Committee, “Gaetano Martino” University Hospital of Messina, Italy. The patients/participants provided their written informed consent to participate in this study.

## Author contributions

GM and AC made conception and design, FB, NM performed acquisition of data. GM, AC, AG, NM made analysis and interpretation of data. GM and AC drafted the article. CV, FC, GS, TL-J, PS and GC revised it critically for important intellectual content. All authors contributed to the article and approved the submitted version.
